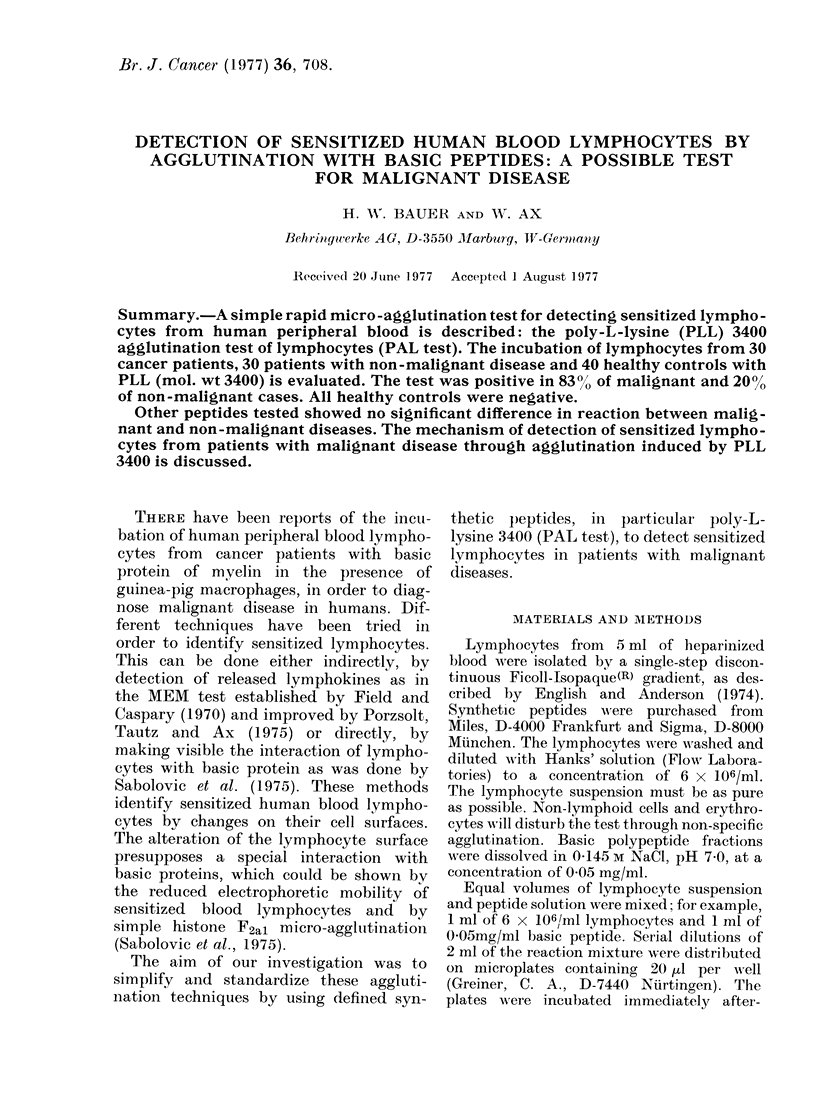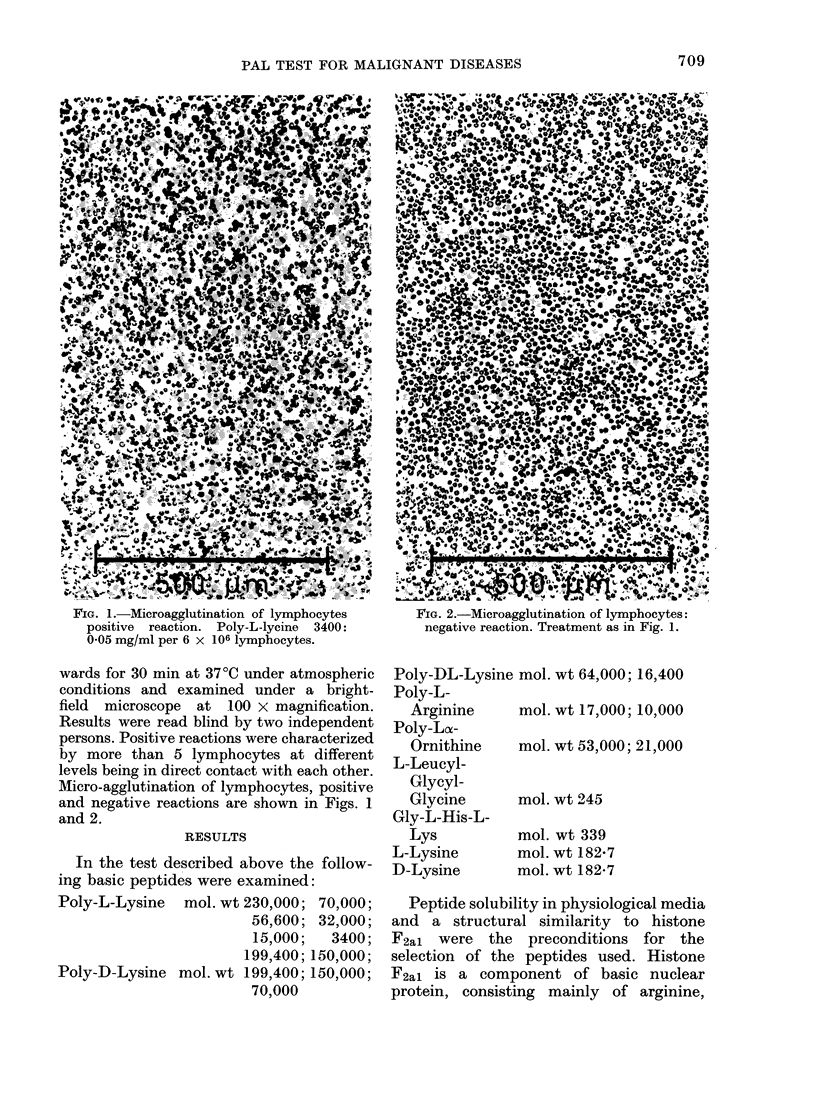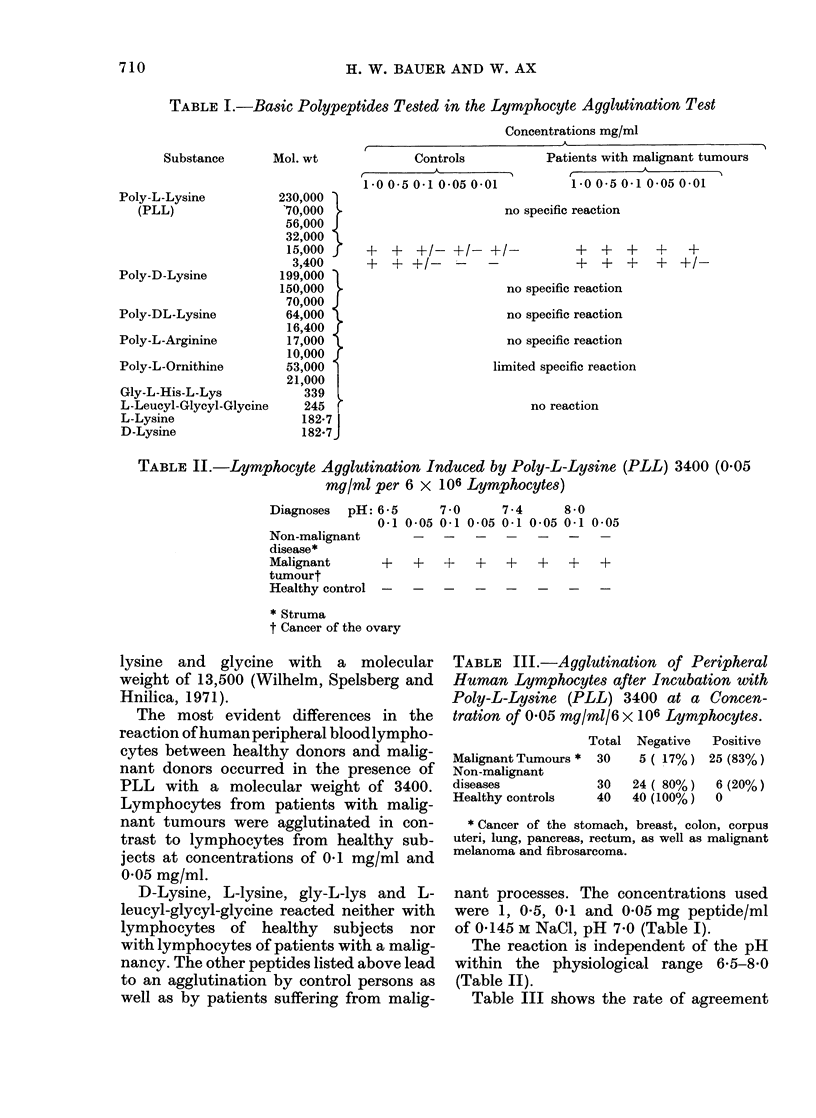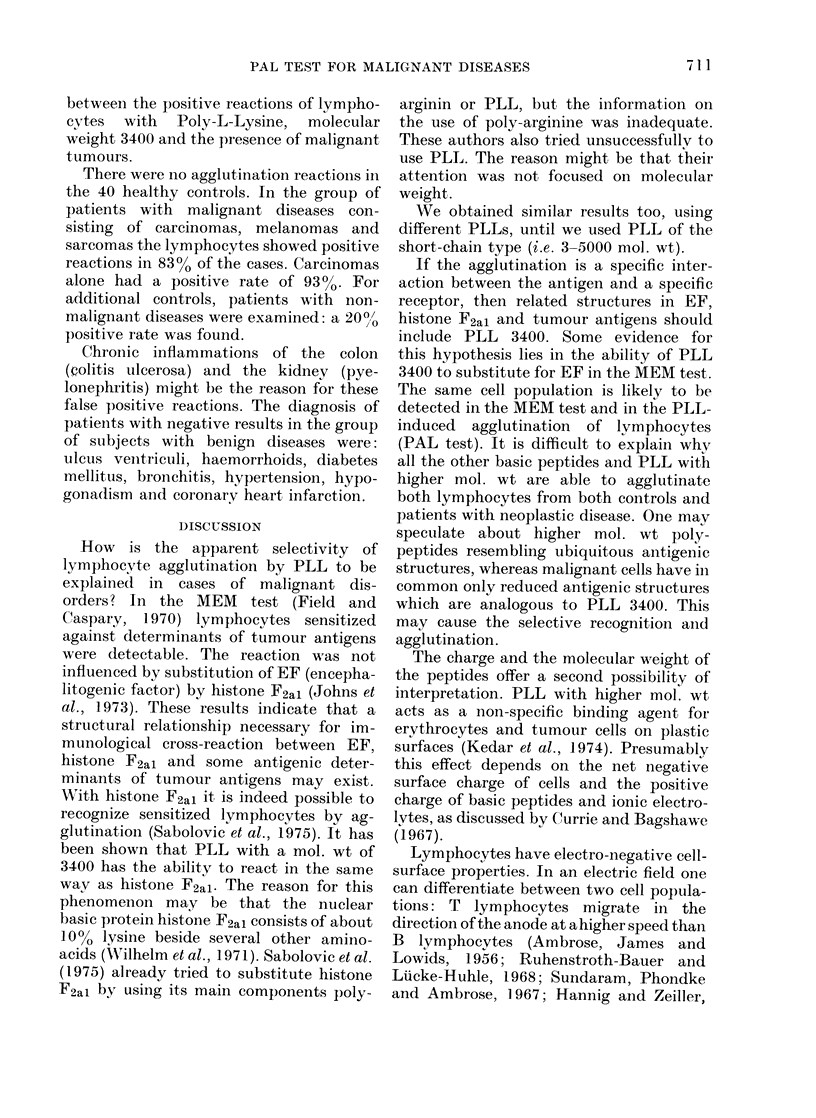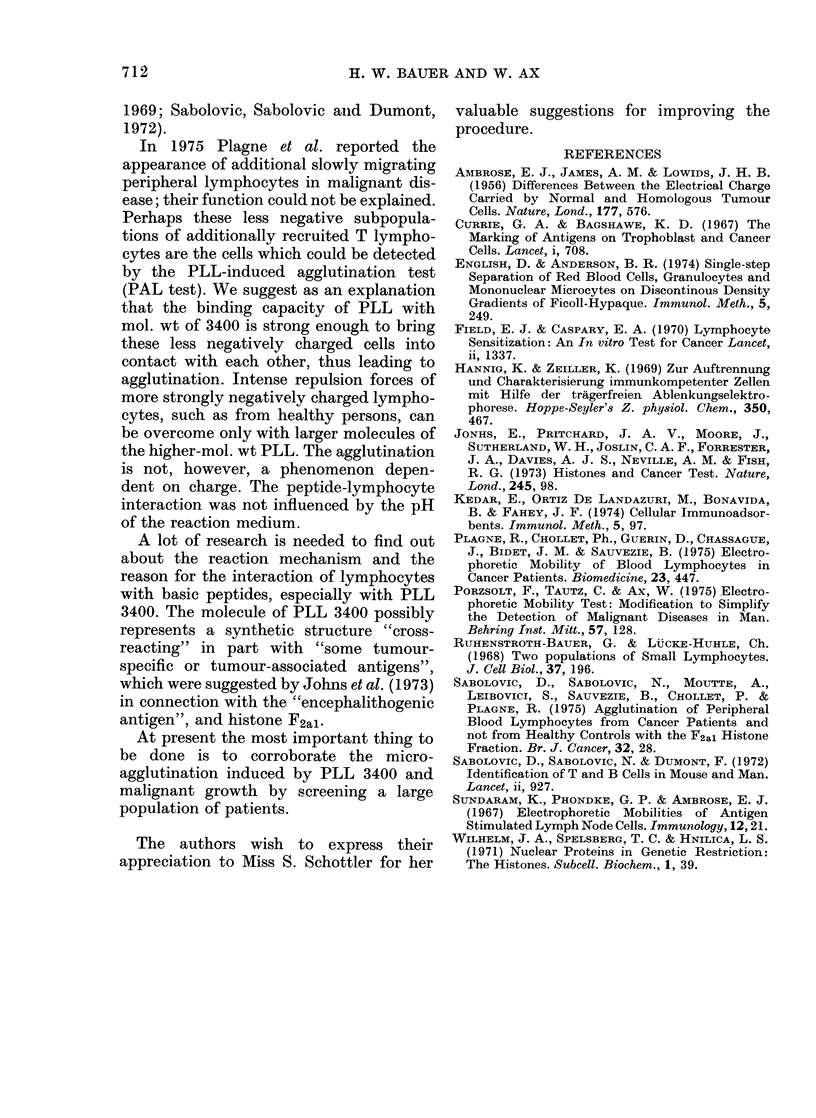# Detection of sensitized human blood lymphocytes by agglutination with basic peptides: a possible test for malignant disease.

**DOI:** 10.1038/bjc.1977.253

**Published:** 1977-12

**Authors:** H. W. Bauer, W. Ax

## Abstract

**Images:**


*Br. J. Cancer* (1977) **36**, 708.

### DETECTION OF SENSITIZED HUMAN BLOOD LYMPHOCYTES BY AGGLUTINATION WITH BASIC PEPTIDES: A POSSIBLE TEST FOR MALIGNANT DISEASE

H. W. BAUER AND W. AX

*Behringwerke AG, D-3550 Marburg, W-Germany*

Received 20 June 1977 Accepted 1 August 1977

**Summary.—A simple rapid micro-agglutination test for detecting sensitized lymphocytes from human peripheral blood is described: the poly-L-lysine (PLL) 3400 agglutination test of lymphocytes (PAL test). The incubation of lymphocytes from 30 cancer patients, 30 patients with non-malignant disease and 40 healthy controls with PLL (mol. wt 3400) is evaluated. The test was positive in 83% of malignant and 20% of non-malignant cases. All healthy controls were negative.**

**Other peptides tested showed no significant difference in reaction between malignant and non-malignant diseases. The mechanism of detection of sensitized lymphocytes from patients with malignant disease through agglutination induced by PLL 3400 is discussed.**

THERE have been reports of the incubation of human peripheral blood lymphocytes from cancer patients with basic protein of myelin in the presence of guinea-pig macrophages, in order to diagnose malignant disease in humans. Different techniques have been tried in order to identify sensitized lymphocytes. This can be done either indirectly, by detection of released lymphokines as in the MEM test established by Field and Caspary (1970) and improved by Porzsolt, Tautz and Ax (1975) or directly, by making visible the interaction of lymphocytes with basic protein as was done by Sabolovic *et al.* (1975). These methods identify sensitized human blood lymphocytes by changes on their cell surfaces. The alteration of the lymphocyte surface presupposes a special interaction with basic proteins, which could be shown by the reduced electrophoretic mobility of sensitized blood lymphocytes and by simple histone $F_{2a1}$ micro-agglutination (Sabolovic *et al.*, 1975).

The aim of our investigation was to simplify and standardize these agglutination techniques by using defined synthetic peptides, in particular poly-L-lysine 3400 (PAL test), to detect sensitized lymphocytes in patients with malignant diseases.

### MATERIALS AND METHODS

Lymphocytes from 5 ml of heparinized blood were isolated by a single-step discontinuous Ficoll-Isopaque$^{(R)}$ gradient, as described by English and Anderson (1974). Synthetic peptides were purchased from Miles, D-4000 Frankfurt and Sigma, D-8000 München. The lymphocytes were washed and diluted with Hanks' solution (Flow Laboratories) to a concentration of $6 \times 10^6$/ml. The lymphocyte suspension must be as pure as possible. Non-lymphoid cells and erythrocytes will disturb the test through non-specific agglutination. Basic polypeptide fractions were dissolved in 0·145 M NaCl, pH 7·0, at a concentration of 0·05 mg/ml.

Equal volumes of lymphocyte suspension and peptide solution were mixed; for example, 1 ml of $6 \times 10^6$/ml lymphocytes and 1 ml of 0·05mg/ml basic peptide. Serial dilutions of 2 ml of the reaction mixture were distributed on microplates containing 20 $\mu$l per well (Greiner, C. A., D-7440 Nürtingen). The plates were incubated immediately after-

FIG. 1.—Microagglutination of lymphocytes positive reaction. Poly-L-lycine 3400: 0·05 mg/ml per 6 × $10^6$ lymphocytes.

FIG. 2.—Microagglutination of lymphocytes: negative reaction. Treatment as in Fig. 1.

wards for 30 min at 37 °C under atmospheric conditions and examined under a bright-field microscope at 100 × magnification. Results were read blind by two independent persons. Positive reactions were characterized by more than 5 lymphocytes at different levels being in direct contact with each other. Micro-agglutination of lymphocytes, positive and negative reactions are shown in Figs. 1 and 2.

RESULTS

In the test described above the following basic peptides were examined:

| | |
|---|---|
| Poly-L-Lysine | mol. wt 230,000; 70,000; 56,600; 32,000; 15,000; 3400; 199,400; 150,000; |
| Poly-D-Lysine | mol. wt 199,400; 150,000; 70,000 |
| Poly-DL-Lysine | mol. wt 64,000; 16,400 |
| Poly-L-Arginine | mol. wt 17,000; 10,000 |
| Poly-Lα-Ornithine | mol. wt 53,000; 21,000 |
| L-Leucyl-Glycyl-Glycine | mol. wt 245 |
| Gly-L-His-L-Lys | mol. wt 339 |
| L-Lysine | mol. wt 182·7 |
| D-Lysine | mol. wt 182·7 |

Peptide solubility in physiological media and a structural similarity to histone $F_{2a1}$ were the preconditions for the selection of the peptides used. Histone $F_{2a1}$ is a component of basic nuclear protein, consisting mainly of arginine,

TABLE I.—*Basic Polypeptides Tested in the Lymphocyte Agglutination Test*

| Substance | Mol. wt | Concentrations mg/ml: Controls 1·0 | 0·5 | 0·1 | 0·05 | 0·01 | Patients with malignant tumours 1·0 | 0·5 | 0·1 | 0·05 | 0·01 |
|---|---|---|---|---|---|---|---|---|---|---|---|
| Poly-L-Lysine (PLL) | 230,000, 70,000, 56,000 | no specific reaction | | | | | | | | | |
| | 32,000, 15,000 | + | + | +/− | +/− | +/− | + | + | + | + | + |
| | 3,400 | + | + | +/− | − | − | + | + | + | + | +/− |
| Poly-D-Lysine | 199,000, 150,000, 70,000 | no specific reaction | | | | | | | | | |
| Poly-DL-Lysine | 64,000, 16,400 | no specific reaction | | | | | | | | | |
| Poly-L-Arginine | 17,000, 10,000 | no specific reaction | | | | | | | | | |
| Poly-L-Ornithine | 53,000, 21,000 | limited specific reaction | | | | | | | | | |
| Gly-L-His-L-Lys | 339 | no reaction | | | | | | | | | |
| L-Leucyl-Glycyl-Glycine | 245 | | | | | | | | | | |
| L-Lysine | 182·7 | | | | | | | | | | |
| D-Lysine | 182·7 | | | | | | | | | | |

TABLE II.—*Lymphocyte Agglutination Induced by Poly-L-Lysine (PLL)* 3400 (0·05 *mg/ml per* 6 × 10⁶ *Lymphocytes*)

| Diagnoses | pH: 6·5 0·1 | 6·5 0·05 | 7·0 0·1 | 7·0 0·05 | 7·4 0·1 | 7·4 0·05 | 8·0 0·1 | 8·0 0·05 |
|---|---|---|---|---|---|---|---|---|
| Non-malignant disease* | | − | − | − | − | − | − | − |
| Malignant tumour† | + | + | + | + | + | + | + | + |
| Healthy control | − | − | − | − | − | − | − | − |

* Struma

† Cancer of the ovary lysine and glycine with a molecular weight of 13,500 (Wilhelm, Spelsberg and Hnilica, 1971).

The most evident differences in the reaction of human peripheral blood lymphocytes between healthy donors and malignant donors occurred in the presence of PLL with a molecular weight of 3400. Lymphocytes from patients with malignant tumours were agglutinated in contrast to lymphocytes from healthy subjects at concentrations of 0·1 mg/ml and 0·05 mg/ml.

D-Lysine, L-lysine, gly-L-lys and L-leucyl-glycyl-glycine reacted neither with lymphocytes of healthy subjects nor with lymphocytes of patients with a malignancy. The other peptides listed above lead to an agglutination by control persons as well as by patients suffering from malignant processes. The concentrations used were 1, 0·5, 0·1 and 0·05 mg peptide/ml of 0·145 M NaCl, pH 7·0 (Table I).

TABLE III.—*Agglutination of Peripheral Human Lymphocytes after Incubation with Poly-L-Lysine (PLL)* 3400 *at a Concentration of* 0·05 *mg/ml/*6 × 10⁶ *Lymphocytes.*

| | Total | Negative | Positive |
|---|---|---|---|
| Malignant Tumours * | 30 | 5 ( 17%) | 25 (83%) |
| Non-malignant diseases | 30 | 24 ( 80%) | 6 (20%) |
| Healthy controls | 40 | 40 (100%) | 0 |

* Cancer of the stomach, breast, colon, corpus uteri, lung, pancreas, rectum, as well as malignant melanoma and fibrosarcoma.

The reaction is independent of the pH within the physiological range 6·5–8·0 (Table II).

Table III shows the rate of agreement between the positive reactions of lymphocytes with Poly-L-Lysine, molecular weight 3400 and the presence of malignant tumours.

There were no agglutination reactions in the 40 healthy controls. In the group of patients with malignant diseases consisting of carcinomas, melanomas and sarcomas the lymphocytes showed positive reactions in 83% of the cases. Carcinomas alone had a positive rate of 93%. For additional controls, patients with non-malignant diseases were examined: a 20% positive rate was found.

Chronic inflammations of the colon (colitis ulcerosa) and the kidney (pyelonephritis) might be the reason for these false positive reactions. The diagnosis of patients with negative results in the group of subjects with benign diseases were: ulcus ventriculi, haemorrhoids, diabetes mellitus, bronchitis, hypertension, hypogonadism and coronary heart infarction.

### DISCUSSION

How is the apparent selectivity of lymphocyte agglutination by PLL to be explained in cases of malignant disorders? In the MEM test (Field and Caspary, 1970) lymphocytes sensitized against determinants of tumour antigens were detectable. The reaction was not influenced by substitution of EF (encephalitogenic factor) by histone $F_{2a1}$ (Johns *et al.*, 1973). These results indicate that a structural relationship necessary for immunological cross-reaction between EF, histone $F_{2a1}$ and some antigenic determinants of tumour antigens may exist. With histone $F_{2a1}$ it is indeed possible to recognize sensitized lymphocytes by agglutination (Sabolovic *et al.*, 1975). It has been shown that PLL with a mol. wt of 3400 has the ability to react in the same way as histone $F_{2a1}$. The reason for this phenomenon may be that the nuclear basic protein histone $F_{2a1}$ consists of about 10% lysine beside several other amino-acids (Wilhelm *et al.*, 1971). Sabolovic *et al.* (1975) already tried to substitute histone $F_{2a1}$ by using its main components poly-arginin or PLL, but the information on the use of poly-arginine was inadequate. These authors also tried unsuccessfully to use PLL. The reason might be that their attention was not focused on molecular weight.

We obtained similar results too, using different PLLs, until we used PLL of the short-chain type (*i.e.* 3–5000 mol. wt).

If the agglutination is a specific interaction between the antigen and a specific receptor, then related structures in EF, histone $F_{2a1}$ and tumour antigens should include PLL 3400. Some evidence for this hypothesis lies in the ability of PLL 3400 to substitute for EF in the MEM test. The same cell population is likely to be detected in the MEM test and in the PLL-induced agglutination of lymphocytes (PAL test). It is difficult to explain why all the other basic peptides and PLL with higher mol. wt are able to agglutinate both lymphocytes from both controls and patients with neoplastic disease. One may speculate about higher mol. wt polypeptides resembling ubiquitous antigenic structures, whereas malignant cells have in common only reduced antigenic structures which are analogous to PLL 3400. This may cause the selective recognition and agglutination.

The charge and the molecular weight of the peptides offer a second possibility of interpretation. PLL with higher mol. wt acts as a non-specific binding agent for erythrocytes and tumour cells on plastic surfaces (Kedar *et al.*, 1974). Presumably this effect depends on the net negative surface charge of cells and the positive charge of basic peptides and ionic electrolytes, as discussed by Currie and Bagshawe (1967).

Lymphocytes have electro-negative cell-surface properties. In an electric field one can differentiate between two cell populations: T lymphocytes migrate in the direction of the anode at a higher speed than B lymphocytes (Ambrose, James and Lowids, 1956; Ruhenstroth-Bauer and Lücke-Huhle, 1968; Sundaram, Phondke and Ambrose, 1967; Hannig and Zeiller,

1969; Sabolovic, Sabolovic and Dumont, 1972).

In 1975 Plagne *et al.* reported the appearance of additional slowly migrating peripheral lymphocytes in malignant disease; their function could not be explained. Perhaps these less negative subpopulations of additionally recruited T lymphocytes are the cells which could be detected by the PLL-induced agglutination test (PAL test). We suggest as an explanation that the binding capacity of PLL with mol. wt of 3400 is strong enough to bring these less negatively charged cells into contact with each other, thus leading to agglutination. Intense repulsion forces of more strongly negatively charged lymphocytes, such as from healthy persons, can be overcome only with larger molecules of the higher-mol. wt PLL. The agglutination is not, however, a phenomenon dependent on charge. The peptide-lymphocyte interaction was not influenced by the pH of the reaction medium.

A lot of research is needed to find out about the reaction mechanism and the reason for the interaction of lymphocytes with basic peptides, especially with PLL 3400. The molecule of PLL 3400 possibly represents a synthetic structure "cross-reacting" in part with "some tumour-specific or tumour-associated antigens", which were suggested by Johns *et al.* (1973) in connection with the "encephalithogenic antigen", and histone $F_{2a1}$.

At present the most important thing to be done is to corroborate the micro-agglutination induced by PLL 3400 and malignant growth by screening a large population of patients.

The authors wish to express their appreciation to Miss S. Schottler for her valuable suggestions for improving the procedure.

REFERENCES

AMBROSE, E. J., JAMES, A. M. & LOWIDS, J. H. B. (1956) Differences Between the Electrical Charge Carried by Normal and Homologous Tumour Cells. *Nature, Lond.*, **177**, 576.

CURRIE, G. A. & BAGSHAWE, K. D. (1967) The Marking of Antigens on Trophoblast and Cancer Cells. *Lancet*, i, 708.

ENGLISH, D. & ANDERSON, B. R. (1974) Single-step Separation of Red Blood Cells, Granulocytes and Mononuclear Microcytes on Discontinous Density Gradients of Ficoll-Hypaque. *Immunol. Meth.*, **5**, 249.

FIELD, E. J. & CASPARY, E. A. (1970) Lymphocyte Sensitization: An *In vitro* Test for Cancer *Lancet*, ii, 1337.

HANNIG, K. & ZEILLER, K. (1969) Zur Auftrennung und Charakterisierung immunkompetenter Zellen mit Hilfe der trägerfreien Ablenkungselektrophorese. *Hoppe-Seyler's Z. physiol. Chem.*, **350**, 467.

JONHS, E., PRITCHARD, J. A. V., MOORE, J., SUTHERLAND, W. H., JOSLIN, C. A. F., FORRESTER, J. A., DAVIES, A. J. S., NEVILLE, A. M. & FISH, R. G. (1973) Histones and Cancer Test. *Nature, Lond.*, **245**, 98.

KEDAR, E., ORTIZ DE LANDAZURI, M., BONAVIDA, B. & FAHEY, J. F. (1974) Cellular Immunoadsorbents. *Immunol. Meth.*, **5**, 97.

PLAGNE, R., CHOLLET, Ph., GUERIN, D., CHASSAGUE, J., BIDET, J. M. & SAUVEZIE, B. (1975) Electrophoretic Mobility of Blood Lymphocytes in Cancer Patients. *Biomedicine*, **23**, 447.

PORZSOLT, F., TAUTZ, C. & AX, W. (1975) Electrophoretic Mobility Test: Modification to Simplify the Detection of Malignant Diseases in Man. *Behring Inst. Mitt.*, **57**, 128.

RUHENSTROTH-BAUER, G. & LÜCKE-HUHLE, Ch. (1968) Two populations of Small Lymphocytes. *J. Cell Biol.*, **37**, 196.

SABOLOVIC, D., SABOLOVIC, N., MOUTTE, A., LEIBOVICI, S., SAUVEZIE, B., CHOLLET, P. & PLAGNE, R. (1975) Agglutination of Peripheral Blood Lymphocytes from Cancer Patients and not from Healthy Controls with the $F_{2a1}$ Histone Fraction. *Br. J. Cancer*, **32**, 28.

SABOLOVIC, D., SABOLOVIC, N. & DUMONT, F. (1972) Identification of T and B Cells in Mouse and Man. *Lancet*, ii, 927.

SUNDARAM, K., PHONDKE, G. P. & AMBROSE, E. J. (1967) Electrophoretic Mobilities of Antigen Stimulated Lymph Node Cells. *Immunology*, **12**, 21.

WILHELM, J. A., SPELSBERG, T. C. & HNILICA, L. S. (1971) Nuclear Proteins in Genetic Restriction: The Histones. *Subcell. Biochem.*, **1**, 39.